# A dynamic approach to communication in health literacy education

**DOI:** 10.1186/s12909-016-0785-z

**Published:** 2016-10-21

**Authors:** Herman Veenker, Wolter Paans

**Affiliations:** 1 Hanze University of Applied Sciences, P.O. Box 70030, 9704 AA Groningen, Netherlands; 2 Zernikeplein 9, 9747 AS Groningen, Netherlands; 3 Hanze University of Applied Sciences, Eyssoniusplein 18, 9714 CE Groningen, Netherlands

**Keywords:** Health literacy, SDT, Autonomy, Curriculum development for medical students and practitioners, Interaction

## Abstract

**Background:**

Research within the framework of Self-Determination Theory (SDT) indicates that patients' autonomy is to be considered a critical health care outcome in its own right since it promotes improved mental and physical health.

This paper presents an analysis of studies addressing communication and interaction interventions in health literacy curricula for medical and health care practitioners, focusing on patient-oriented skills in “making sense” and “to adapt and self-manage”. For evaluating interventions, underlying communication models were traced. The criteria for good practice are “making sense” and “supporting autonomy in making choices”. For the search of interventions, keywords from both the framework of the EU-project, Intervention Research on Health Literacy among Ageing population (IROHLA (The IROHLA project received financial support from the European Union through FP7 Grant 305831)), as well as the SDT (Self Determination Theory) were applied.

The research question of this paper is to what degree is autonomy supporting communication skills part of the curricula of health literacy (HL) for medical and health care practitioners and providers? A Pubmed search revealed: a) that “making sense” is clearly represented in HL interventions in curricula; however, b) very few interventions teach medical and health care practitioners how to give autonomy support in the interaction with their (future) patients.

Four promising, beneficial practices were identified. Several recommendations were presented encouraging curriculum developers to adapt skills of supporting autonomy into their programs.

**Methods:**

A qualitative content analysis of interventions in the curricula of communication and interaction skills for medical students and practitioners.

**Results:**

A review of literature indicates: a) most interventions in curricula for medical students and practitioners are focusing on skills in adequately providing information to patients by using an underlying (advanced) Sender-Message-Receiver Model; and b) only a few interventions in curricula are available for providing the acquisition of interaction skills in supporting autonomy.

**Conclusions:**

The proposal of Huber and others to change the emphasis in the definition of the WHO definition on health towards “to adapt and self manage” has impact on the training of medical students and practioners in dealing with patients with low levels of health literacy. From the present study it can be concluded that a dynamic approach to communication can be linked to theoretical constructs on self-management. In such an approach interaction techniques like scaffolding can increase the level of HL of the patient.

## Background

Health literacy (HL) is one of the social determinants of health and reflects how well individuals can understand, assimilate, and critically reflect on information with regard to health and illness. Health literacy is a critical condition to improve mental and physical health. The IROHLA project aims at innovating the conceptual understanding of health literacy interventions in Europe. Tackling health literacy problems in the ageing population leds to social innovation and leads to reduction of costs of healthcare [2]. In EU countries, 10 %–30 % of the population has insufficient health literacy skills which is associated with higher morbidity and mortality while utilization of health services is higher, and treatment outcomes are more unsatisfactory than average. Approximately 12 % of the population in Europe has inadequate health literacy competencies and 35 % have problematic health literacy competencies. The issue is more serious in the aging population even though addressing health literacy problems in the aging population leads to social innovation and the reduction of the costs of healthcare. With respect to social innovation, it is relevant to note that the Irohla-project notes that “there is an association between the levels of health literacy and the self assessed health status in the population. Higher levels of health literacy go hand in hand with higher self assessed health status. Low health literacy is associated with lower perceived health status. These findings confirm that health literacy is key priority for improving health of senior citizens in Europe. In this age group the health literacy related problems are relatively high and the perceived health status is relatively low” [1–3].

The Irohla project investigates health literature interventions among the ageing population and will investigate in stakeholders.

As an institute for the education and training of health care professionals and as a partner of the International EU- project Irohla (International Research on Health Literacy) [2], the Hanze University of Applied Sciences (HUAS) is interested in curriculum development for health care practitioners.

The central aim of HUAS in the IROHLA-project is to define, analyze, and search for beneficial practices in an HL curriculum development for medical and health care practitioners. First, a number of commonly used definitions on health literacy will be analyzed into its main components, second we will elaborate on these components and compare them with modern models of communication. Third, we will use these models of communication as a criterion for detecting promising interventions on curriculum development for health care practitioners.

### Health literacy definitions

An important issue in selecting good communication and interaction practices is obtaining relevant criteria for evaluation purposes. Based on theoretical and conceptual studies, it is known that self-assessment, self-regulating, and self-management are important aspects of social innovation [4]. For that reason, our approach is to utilize theoretical constructs linked to self-management for the evaluation of good practices. This position is being supported by recent criticism on the WHO definition of health as “complete wellbeing”. Several researchers and policy makers claim that this part of the definition is no longer valid considering the increase in chronic diseases. Huber and colleagues propose changing the emphasis towards “the ability to adapt and self-manage” in the face of social, physical, and emotional challenges [5].

Taking the extensive number of definitions on health *literacy* definitions into consideration, it can be determined that most definitions consist of two components; one part contains information and “making sense” and the other is on understanding and using information aiming at “making choices”. The second part also includes the element of self-management (Table 1).

**Table 1:** A small experpt of the variety of definitions of healthy literacy

“Health literacy represents the cognitive and social skills which determine the motivation and ability of individuals to gain access to, understand and use information in ways which promote and maintain good health. Health literacy means more than being able to read pamphlets and successfully make appointments. By improving people’s access to health information and their capacity to use it effectively, health literacy is critical to empowerment” ([18], p264).
“The wide range of skills and competencies that people develop to seek out, comprehend, evaluate and use health information and concepts to make informed choices, reduce health risks and increase quality of life” ([26], p196–197).
“The degree to which individuals have the capacity to obtain, process and understand basic health information and services needed to make appropriate health decisions” [27].
“The capacity to obtain, process, and understand basic health information and services needed to make appropriate health decisions” [10–13], p795, [27–30], [31, 32].
“Health literacy is the ability of patients to obtain, understand, and use medical information to benefit their health and to navigate through the health care system” [33].

### Models of communication

**Fig. 1:**
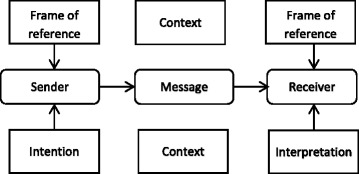
the classic communication model (middle section), dedicated with personalized and contextual factors modeling advanced versions (upper and lower section). For the purpose of the schematic representation, the channel as well as a feedback loop are omitted

Since the focus of all of the definitions is in regard to information, a more thorough inspection on information models can clarify the structure of interventions. The idea is that interventions can be analyzed by matching an information model to an intervention. Information models have gradually evolved from the classical, unidirectional (Berlo, 1960) [6] to advanced models (Fig. 1) and towards complex models that incorporate dynamic systems such as the transactional and the constructivist models. The latter also particularly forms an account for self-management and self-regulation. Thus, the evaluation of the interventions can be analyzed by employing the (implicit) communication model, varying from the classical communication model to advanced models (including the upper and lower sections in combination with the middle section of Fig. 1) and, finally, to models that closely correspond to a modern definition for health such as transactional and constructivist communication models (Fig. 1).

An important characteristic of transactional, constructivist models of communication is the introduction of a dynamic perspective regarding the elements in the model. These models incorporate constructs that facilitate the autonomy of the patient. According to a dynamic view, the “message” is emergent; it emerges as an outcome of negotiation on meaning. Briefly stated, the message is not static as it is in classical models but is fluid and emerges in the interaction.

Examples of health problems that have a high demand on the ability to adapt and self-manage are often related to lifestyle or to decisions that require weighing of benefits and harm between options and lifestyle such as the mode of birth delivery, breast cancer surgery, location of care at the end of life, obesity and participation in a weight loss program, adherence to medication prescriptions [7], coping with cardiovascular diseases, blood sugar monitoring and diabetes [8], smoking cessation [9] and engaging in more physical activity [1, 10] and the like.

**Fig. 2:**
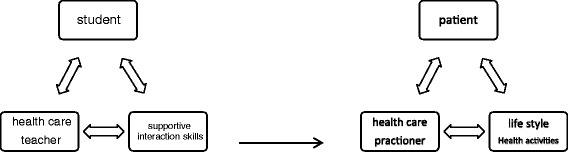
Basic scheme for transactional and constructivist models

**Fig. 3:**
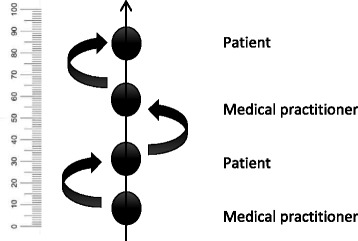
the scaffolding of higher levels of autonomy on a fictional scale 0- 100

Figure 2 provides the basic scheme for transactional and constructivist models. These figures express that knowledge and skills emerge in a dynamic triangle [11]. All of the elements in the model are vitally related to each other. The left side of Fig. 2 represents the student in the curriculum which is the focus of this paper. The right side of Fig. 3 represents the professional context of health care.

The dynamic triangle on acquisition (left side of Fig. 2) and providing supportive autonomy interactions (right side of Fig. 2). Each element interacts with each other in an emerging process of negotiation on meaning. In this perspective, information is considered as an emerging process and not as a static construct of concepts.

Since all of the elements in the model are effectively related to each other, all elements can change. In a dynamic view, changes occur over several time scales. The smallest time scale is at the micro genetic level, the level of face-to-face-interaction itself. At this level, utterances can be transcribed and coded for analysis. Proximal variables can be made visible.

Figure 3 expresses a bidirectional process in which the development of autonomy of the patient can help the health practitioner to improve autonomy-supportive communication, as in Fig. 2 (right side). If this coupling can be made, a positive upward spiral will emerge [11]. Fundamental are the interaction skills, such as the scaffolding of the health practitioner, that are required to create such an autonomy spiral. Importantly, the role of the health care practitioner obtains a new dimension. The practitioner also becomes an expert in providing support in a specific domain and autonomy in such a way that the patient and the practitioner both become involved in a long-term process of *learning*.

### Self-determination theory as a theoretical framework for searching good practices

Self Determination Theory, referred to as SDT, [12] was selected as the theoretical framework since it is a theory on motivation that incorporates key constructs like autonomy, competence, and relatedness; concepts that precisely explain behavior that is required for the faculty of “to adapt and self-manage”.

SDT is a widely accepted theory in social and behavior disciplines (including sports, pedagogy, psychology, and education). A meta-analysis of Ng et al (2012) [1] examined the hypotheses that behavior change is more effective and enduring when patients are autonomously motivated. Ng et al (2012) [1] identified 184 SDT-based studies in the health domain with independent data sets. The research group reports that “the observed effect sizes were moderate in most cases, and the overall pattern was in accordance with SDT”.

Competence, autonomy, and relatedness as well as autonomous self-regulation “predicted moderate to strong levels of patient welfare, such as better mental health and higher levels of health behaviors that are linked to physical health and length of life”. “Together, SDT constructs predicted important outcomes across the biophysical continuum in systems theory (..)”. These findings indicate that health literacy is conditional to promote patients’ autonomy, which is now considered a critical health care outcome in its own right, also promotes improved mental and physical health.

The research question of this paper is to what degree is autonomy supporting communication skills part of the curricula of health literacy (HL) for medical and health care practitioners and providers?

## Methods

This section deals with the search for beneficial practices in an health literacy curriculum development for medical and health care practitioners.

Within the IROHLA project, a set of MeSH terms and search keys for communication and interaction studies was explored whereby two options emerged [13]. The first option is to trace interventions and decide what are effective factors based on quantitative analyses of the interventions or, alternatively, good practices can be ascertained by employing qualitative criteria that are suitable as robust theories.

After a first scan based on titles and abstracts of intervention studies, it became evident that the quantitative, statistical analysis of this primary corpus (*n* = 250 interventions) was not feasible because of the diversity in the research designs. Alternatively, to reduce and specify the corpus, we used: a) health literacy definitions, b) models of communication, and c)the self-determination theory [1, 7, 14]

Two separate search rounds were conducted as show in Table 2. In the first round, we searched using MeSH terms in the Medline database as shown in Table 2 left column. I the second case, we searched on Self-Determination theory…

**Table 2:**
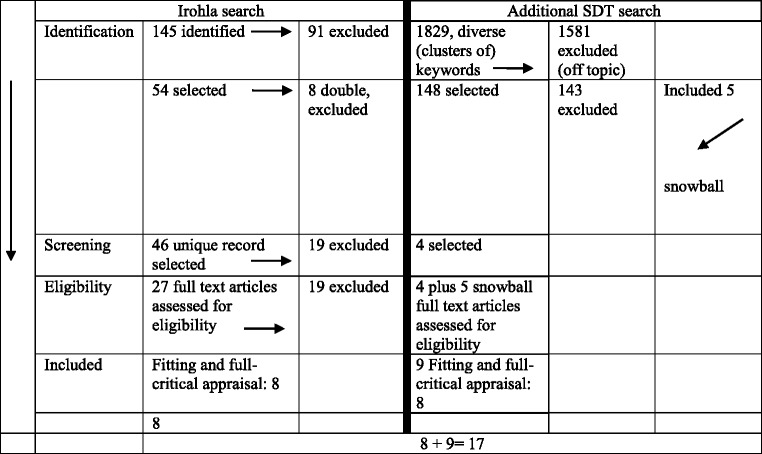
A Prisma flow chart on the search process

1. A search employing key words (MeSH terms) for a search in the Medline database is provided in Table 2, left column.
2. A search on the Self-Determination Theory (Table 2, right column) also in the Medline database using MeSH codes. After it became evident that the MeSH codes were not sufficient due to the fact that there is no specific MeSH code for the Self-Determination Theory, a search was conducted with the search terms listed. In Table 3 specific searches, the number of hits and number of targets are represented. Hits correspond to the selected term, however, do not necessarily accord with the research question. Targets based on the relevance of the contents of the full paper, however, are in agreement with the research question. In Table 2 a flow chart derived from the Prisma model [15] the search process is being depicted.

**Table 3:** Search terms with specific searches, number of hits, and number of targets

Search terms	Hits and targets
health literacy education	6378 hits; narrowed down, c.f. 2. and 3.
health literacy education professionals: Importantly, the step to link health literacy to SDT failed:	1143 hits; no targets
SDT and health literacy: This motivated to new searches using a diversity of terms linked to self-determination theory	0 hits
health literacy education self-efficacy	254 hits, no targets
motivation theory health interventions	783 hits; narrowed down (c.f. 5.)
motivation theory communication skills	127 hits, no targets (1 off topic)
motivation theory health interventions curricula	13 hits, no targets
health literacy education trainees	23 hits, no targets
SDT Health Care	17 hits, no targets
SDT professionals health car	8 hits, no targets, 2 snowball papers
SDT health care	1 target, 49 hits
SDT theory communication skills health professionals	40 hits, no targets
SDT training professionals health	0 hits, no targets
SDT skills in health curricula	0 hits, no targets
SDT health literacy education	0 hits, no targets
Interaction skills health literacy	1 target, 57 hits, 2 papers for snowball search
scaffolding health education	42 hits, no targets
scaffolding skills health professionals	4 hits, no targets
scaffolding skills health education	1 target, 16 hits
scaffolding health literacy	No targets, 1 double hit already counted
scaffolding skills health workers	0 hits, no targets
health literacy education trainees	23 hits, no targets
communication skills students in health literacy	1 target, 13 hits
	(1 snowball via expert)

A search string for finding most (not all) of the interventions:

(“Health Literacy”[Mesh] OR “Health Literacy”[Title/Abstract]) AND (“Communication”[Mesh] OR “Communication"[Title/Abstract] OR “Curriculum”[Mesh] OR “Curriculum”[Title/Abstract] OR “Curricula”[Title/Abstract] OR “Students”[Mesh] OR “Student”[Title/Abstract] OR “Students”[Title/Abstract]) AND (English[lang]) AND (“2006/01/01”[PDAT] : “2016/12/31”[PDAT])

## Results

The results are depicted in Table 4.

**Table 4:** Summary of Pubmed search on communication models in interventions of curricula HL

Authors	Keywords and citation	Purpose of the intervention	Intervention approach or strategy	Sample & Evaluation method/	Communication model
	(e.g. Huber, intrinsic motivation, SDT, self-efficacy, Health Literacy, Four Habits Model)				
Chen [34]	Health literacy education; (health literacy in student education)	Improve verbal instruction skills of pharmacy students.	Exercise with re-writing assignments targeting people of low health literacy; tools on measuring language difficulty like Flesch-Kincaid tool.	Student pharmacists: *N* = 303 Evaluation questionnaire on perceived satisfaction of participants.	Focus on Sender and Message; information about Receiver; classical +SMR.
Cotugna [35]	Study mentions: ‘problem of self-management skills’ (p. 878)	The purpose of the project is to develop, implement and evaluate a health literacy module for a nutrition education course that would involve students interacting with professionals.	Learning about the problem of health literacy: the outcome goal of the module was to have students produce and present a 3-hour workshop for health care practitioners on the topic of health literacy. Learning by developing a workshop on the topic.	Female professionals: *N* = 33 Evaluation questionnaire on perceived satisfaction of participants on a workshop.	Focus on Sender and Message; information about Receiver; classical SMR.
Doyle [17]	Language difficulty in healthcare	Improving communication between healthcare professionals and patients focusing on writing patient information leaflets (PILs).	Learning to write at the level of the target group using tools on measuring language difficulty like Flesch-Kincaid tool	Medicine students: *n* = 357, physiotherapy students: *n* = 337. Evaluating the PILs: measurement of language complexity with Flesch-scale on readability. The DISCERN tool was being used for measuring student feedback on the learning experience.	Focus on (readability of the) Message: Classical communication Model.
Finset [36]	Four Habits communication and taking the patient perspective/person- centered approach	To communicate with patients on a personal level.	Four Habits Model (Krupat et al, 2006). The model is based on creating empathic opportunities. Some constructs of the model fit with the construct of autonomy in SDT such as face to face interpersonal exchange using sensitivity to patient cues and concerns.	Literature review	With emphasis on the patients’ perspective, changing R (patient) into S (sender); therefore creating RMS; R<>M<>S This approach most likely takes into account the intrinsic motivation and autonomy of the patient (without making these aspects explicit). Advanced SMR model
Goto [18]	Health literacy education; (health literacy professionals)	The training program was designed to help health professionals understand the gap between professional knowledge—including terms and concepts, and the public’s understanding of health and science related information.	Model program by Rudd (c.f.: http://www.hsph.harvard.edu/healthliteracy/overview-2-2/) http://www.hsph.harvard.edu/healthliteracy/) Updated 6 April 2015	Public Health Nurses: *N* = 33 Quantitative data: questionnaires. Qualitative data: interviews and discussions with participants.	The intervention focuses on skills in developing texts and educational materials. Classical SMR model
Grice [32]	“Elicit the patient’s perspective”. In the model patients’ motivation is included as a part of habit 4: “Invest in the end”.	To assess whether student pharmacists' communication skills improved using the Four Habit Model at a College of Pharmacy; focusing on empathy.	Role play in exercising interviews with patients with formative feedback; summative assessment in real interactions with patients	Student pharmacists: *N* = 158 Scoring of interactions with video; analysis of scores	Interaction model that ensures effectiveness on both sides of the interaction
McCleary [19]	Health literacy education; (health literacy student knowledge)	To improve students’ knowledge of health literacy	A hybrid course on Health Literacy, using 16 online course modules and 7 live class meetings for a baccalaureate nursing program (topic: pharmacology).	Nursing students; *N* = 89 Pretest- posttest; items scoring knowledge on HL	Classical SMR model
Patterson [37]	Health literacy communication	To develop and implement an advanced pharmacy practice experience aiming to increase student's awareness of, acceptance of and ability to apply public concepts in pharmaceutical care.	Acquiring information on HL (reading the AMA's Health literacy manual for Clinicians) and actively participate in a community outreach day in a special community.	Pharmacy student’s: *N* = 9 Discussions on several themes (formative) and assessment scores on 5 abilities, no 5 relates to communication: “Refer a patient to community resources as appropriate.	There are no special activities that can explicitly be linked to a distinct communication model. Assessed ability on five links to the classical SMR model.
Planas [38]	Communication skills, scaffolding, self-directed learning	There is a lack of consensus on the essential components of effective pharmacist-patient communication. There is a need for reliable, authentic, and comprehensive assessments of pharmacy students’ communication skills. The objective of the intervention is a) to describe a communication skills development system (CSD), and b) to evaluate the systems’ effectiveness in a clinical communications course.	Implementing a Communication Skills Development (CSD), special for Clinical Communications. Vygotsky’s constructivists approach for scaffolding skill development of students. A web-based environment also supporting video was used for practicing specific skills.	Student participation: *N* = 123 Evaluation of interviews (two rounds) on four criteria (*n* = 123 faculty, self and patient assessments; *n* = 284 peer assessments). Composition of SOAP notes was used as well.	The learning outcomes are dealing with: “a) effective communication while conducting interviews, b) gather and use pertinent information during patient interview to optimize patients’ drug therapy outcomes, c) compose a well-written SOAP note. d) Provide constructive feedback to self and peers (..) to improve communication with patients, e) construct, present, implement and reflect on a plan of action to achieve goals for improved communication with patients”. Advanced SMR-model.
Poirier [39]	Health promotion and literacy	To design, implement, and evaluate a course on health promotion literacy	Students acquired intercultural communication skills in the context of HL. Activities were grouped into 7 clusters, like the exploration of health beliefs, discussing a film, and developing knowledge on HL and getting familiar with instruments to identify HL	Pharmacy students: *N* = 81 Pre-post model using Inventory for assessing the process of cultural competence among healthcare professionals (IAPCC-R) scores.	Developing cultural competences refers to an advanced SMR model
Primack [40]	Health literacy, patient interaction, patient education	To evaluate an innovative, theory-based, educational intervention involving social marketing and health literacy. The intervention aims to train health care providers to deliver care sensitive to the needs of diverse individuals with varying degrees of HL .	Applying theory of social marketing on communication in HL contexts. Developing skills in developing effective patient materials. Brochure development. Key elements of the approach are: considering the background, abilities and desires of a particular group of patients in their effort to “market” a specific health-related outcome to this “target audience” (cf 1. Introduction).	First year medical students: *N* = 147 Pretest-posttest, matching individuals for comparing results in t-test model. Data were derived from questionnaires (“I feel comfortable taking care of a patient of a different race than me”).	Advanced SMR model, focusing on marketing the message
Roberts [41]	Health literacy curriculum, Teach back method	To implement and evaluate a new health literacy curriculum for third year medical students.	Student learned: 1. to define the concept of HL 2. to describe the impact of HL on patient care (..) 3. to identify patients with low HL (..) 4. to use methods for better communication (like Teach Back)	Third year medical students: *N* = 152 1. written evaluation 2. pre-test - post-test questionnaire 3. assessment on discussion board Blackboard 4. score communication skills with standardized patients in teach back 5. extra post-test (two questions	Advanced SMR
Stacey [10]	Nursing curriculum, patient decision support, decision coaching.	To integrate patient decision support into an existing curriculum.	The Ottawa Decision Support Framework (ODSF) focuses on three aspects: decisional needs, decision quality decision support.	Nursing students: *N* = 114 The integration of the ODSF is being guided by the method of Knowledge to Action Process (Graham et al, 2006). The intervention is not based on experimental data.	Advanced SMR (static interpretation of support)
Sullivan [42]	Health promotion access	To describe a teaching-learning strategy in a baccalaureate school of nursing.	Partnering with community agencies to provide nursing students with cultural awareness experiences and refugee health promotion access. literature studies, formative interviews in the communities; making use of informants for obtaining information	Hmong refugee family representatives: *N* = 40 Outcomes from student and for refugee population. Student outcomes: evaluation of a weekly reflective journal; communication and didactic tools that were developed by the student were assessed. Also a presentation of each student was assessed. For the refugees outcomes to be assessed could be e.g. newly learned words, their verbal explanations etc., summarizing their (growth in) understanding of information taught by the students.	cf. evaluation method: advanced SMR model
Scheckel [30]	Self-efficacy, to provide patients education in a broader systems level context	To describe undergraduate nursing students’ experiences of learning and providing patient education	Students reflected on the question: “One of the core responsibilities of nurses is providing patient education. Nursing education courses often include teaching students to provide patient education. Can you tell me of a time during your nursing education, one that stands out to you, that reflects what it meant to learn and provide patient education?” The answers (also including examples of their practices) of the students were interpreted by the authors.	Undergraduate nursing students: *N* = 8 Interpretative phenomenology; Unstructured face-to-face audio-taped interviews revealed communication skills and sensitivity for patient-contexts of students focusing on understanding and instruction	Classical SMR, model; taking the context of patients into account
Shieh [43]	Nurse education, HL, curricular development; self-regulating	To explore undergraduate nursing students’ experiences in caring for patients with low health literacy.	Student wrote an essay linking a definition of HL to their experiences with patients.	Nursing students: *N* = 70. Qualitative analysis of the essays in several rounds; coding with, e.q.: Simplifying information, reinforcing information, giving written information, using demonstration and Teach Back, adopting additional communication strategies, collaborating with experts; changing patient knowledge and behavior, reducing patient emotional strain, feeling positive about the interaction/experience, failing to change the patient,	Coding refers to (advanced) SMR model
Weiss [44]	Health literacy, clear two way communication	To inform professionals on health literacy; information, approaches	1. web based course; 2. promoting Teach Back	Medical students, residents, fellow, physicians, nurses, therapists, social workers and caregivers (*N* = not provided in the article) Short quizzes, health literacy tests and video vignettes are included in a web based module	1. classical SMR; 2. Teach Back: advanced SMR

(Please note that Table 4 is shown at the end of this document due to the fact that it is larger than one A4)

The results can be reported into four themes: the use of constructivist models, the use of classical SMR-models, the aims of the interventions and the used instruments in interventions.

The main result of the analysis of the search is that no clear examples of transactional or *constructivist models* were found. Closest to the Transactional or Constructivist Models is the 4Habits Model. No clear examples of autonomy-supportive models are determined. The 4Habits and ODSF are the most approximate, however, a sharpening or redefinition of the concept of “support” (ODSF) is needed to satisfy this criterion.

A second outcome is that except for one all interventions incorporate an (implicit) *SMR-model*, moreover a small majority of 10 interventions uses an (implicit) advanced SMR-model. Examples of advanced models are the 4Habits Model, Teach back, Communication Skills Development System (CSD), and the Ottawa Decision Support Framework (ODSF).

A third outcome is that most *interventions are aiming* to improve students’ skills in communication and/or information skills and on increasing students’ knowledge on health literacy [16].

Interventions often focus on: a) knowledge of distal factors (characteristics of people with a lower level of literacy and the implications of this); b) communication skills are limited to “how to make the patient understand what I mean”; learning goals are limited to language use, i.e., effectively sending a message that is often limited in information; also focusing on ensuring that “the patient understands what I mean” (e.g., in Teach Back); c) the production of readable texts (flyers, booklets) that are comprehensible for people with a lower level of health literacy (such as Flesch/Flesch–Kincaid readability tests, c.f. Doyle (2012) [17], Goto (2014) [18]; and, finally, d) tools for testing literacy levels (like S-Tofhla).

Fourth, *commonly used instruments* for measuring health literacy are the Rapid Estimate of Adult Literacy in Medicine, (REALM; -R revised) and the Short Test of Functional Health Literacy in Adults (S-OFHLA); also reported are Single Item Literacy Screener (SILS); and Newest Vital Sign (NVS) (c.f. McCleary-Jones, 2012 [19]).

## Discussion

The research question of this paper is to what degree is autonomy supporting communication skills part of the curricula of health literacy (HL) for medical and health care practitioners and providers? The data reveal that all of the interventions explicitly pay attention to the first part of HL- definitions on information; the part that is on “make sense of it”. Remarkably, the second part of the definitions is missing in most interventions.

This can be explained by the finding that most interventions are based on an SMR Model of information. It appears feasible that classical (advanced) SMR Models of communication incorrectly assume that, if patients with limited health literacy better understand health-care information, they can better enhance their self-care ability (see, for instance, McCleary-Jones et al, 2012 ([19], p214). The misunderstanding is that the “making sense” component of health literacy definitions cannot be identified with the “making choices” part. In the context of making choices, the dynamic context of the patient must be taken into consideration. In addressing the issue of health literacy interventions, it can be argued that professionals not only need to focus on health care information but also on supporting the autonomy of their patients.

Such a constructivists’ approach corresponds closely with upcoming definitions of health and health literacy “to adapt and to self-manage” [5] since this may be the key to success in addressing HL, especially in issues that involve life style.

The finding that there is only a minimal curriculum implemented in SDT constructs is, to some degree, remarkable since: a) in the practice of health care practitioners, several examples can be found of interventions that actually utilize (elements of) SDT in several (psycho)pathologically and/or lifestyle related issues [7]. Obviously, practitioners are more proactive than curriculum developers; b) several curricula do use SDT constructs for motivating their own students [20] as commented by Hoffman (2015) [21] for promoting techniques like scaffolding. From this, it can be concluded that, in the field of education and training of medical practitioners, there is sufficient familiarity with SDT constructs per se; what is needed, however, is a renewed perspective of the classical communication model using Huber’s “to adapt and to self-manage” [5] as a starting point for defining learning outcomes in curricula on the training and education of these practitioners.

Finally we would like to end with the point of view that the constructivist approach as in SDT is embedded in an ecological meta-theory [22]. Bronfenbrenner states that individuals develop in nested structures that define the human ecosystem. Such a meta-theory may be important to develop a modern view on the interactions between in the microsystem and the mesosysteem of health care.

### Recommendations

A first recommendation deals with the implementation of the basic principles of SDT in the curriculum [7]. A very useful approach deals with case-based learning using video. Curriculum developers can make a start with the development of a coaching program for students’ interaction skills using scaffolding techniques in supporting autonomy of patients (for finding comparable examples of c.f. Wetzels (2015) [23] on coaching principles in Science & Technology for teachers). The video-taped interactions provide a very powerful tool for students to learn from by learning how to write transcriptions of interactions for analysis with scales of autonomy-support (c.f. examples of tools on the website of SDT). The videotapes added with transcriptions provide very effective materials not only for for creating awareness but also for training verbal and non-verbal skills in supporting autonomy support. From our experience with students in the field of pedagogics students find it very powerful to use observation- tools using videotaped transcriptions of their own interactions. Practical tools can be found at the website of Deci & Ryan at http://www.selfdeterminationtheory.org (see also Ten Cate et al. [7], p970]).

A second recommendation deals with measuring effects of the improved curriculum. Developers can make use of models derived from a systems theory (Engel, 1977) [24] for evaluating and further improving interventions both in curricula and in daily practice of health literacy. It may be beneficial to make use of already gathered experience in the utilization of SDT in the current practices of medical practioners.

A third recommendation is that advanced SMR-models have a fitting potential to incorporate autonomy-supportive skills based on SDT. In particular this is true for the approaches of the 4Habits Model, the Teach Back Method, the Communication Skills Development System (CSD) and the Ottawa Decision Support Framework (ODSF).

Finally the issue of a lower health literacy level is not only concerning adults. In training health care practitioners, special interaction skills in supporting autonomy for children must strongly be emphasized. Pedagogical-didactic strategies like using child-oriented questions and using scaffolding techniques can be useful tools (Wetzels) [23]. From an ecological point of view it may also be very powerful in certain cases to also involve volunteers in creating an autonomy-supportive environment for patients [25].

## Conclusions

HL is a critical condition to improve mental and physical health. Since the classical communication models are static, the level of HL of a person is considered static. This explains why in most curricula much effort is undertaken in “making sense”. However, in a dynamic view the HL of a person is modeled as a dynamic phenomenon. Therefore the HL of a patient can *decrease* or *increase* in the interaction with a health professional. Only by increasing the HL of the patient s/he becomes more competent in “adapt and self managing” his or her health. A dynamic approach of the interaction between health professional and patient models this communication process. Since most interventions in curricula implicitly use a (elaborated) static communication model, we conclude that a dynamic interaction model is relevant for the training of medical students and practioners in dealing with patients with low levels of health literacy. Furthermore it can be concluded that a dynamic approach to communication can be linked to theoretical constructs on self-management. In such an approach interaction techniques like scaffolding can increase the level of HL of the patient, ecologically differentiating between adults and children.
